# Flavoprotein monooxygenases for oxidative biocatalysis: recombinant expression in microbial hosts and applications

**DOI:** 10.3389/fmicb.2014.00025

**Published:** 2014-02-06

**Authors:** Romina D. Ceccoli, Dario A. Bianchi, Daniela V. Rial

**Affiliations:** ^1^Área Biología Molecular, Departamento de Ciencias Biológicas, Facultad de Ciencias Bioquímicas y Farmacéuticas, Universidad Nacional de RosarioCONICET, Rosario, Argentina; ^2^Instituto de Química Rosario (IQUIR, CONICET-UNR), Área Análisis de Medicamentos, Departamento de Química Orgánica, Facultad de Ciencias Bioquímicas y Farmacéuticas, Universidad Nacional de RosarioRosario, Argentina

**Keywords:** flavoprotein monooxygenase, Baeyer–Villiger oxidation, biooxidations, biocatalysis, sulfoxidation, epoxidation, hydroxylation, recombinant biocatalyst

## Abstract

External flavoprotein monooxygenases comprise a group of flavin-dependent oxidoreductases that catalyze the insertion of one atom of molecular oxygen into an organic substrate and the second atom is reduced to water. These enzymes are involved in a great number of metabolic pathways both in prokaryotes and eukaryotes. Flavoprotein monooxygenases have attracted the attention of researchers for several decades and the advent of recombinant DNA technology caused a great progress in the field. These enzymes are subjected to detailed biochemical and structural characterization and some of them are also regarded as appealing oxidative biocatalysts for the production of fine chemicals and valuable intermediates toward active pharmaceutical ingredients due to their high chemo-, stereo-, and regioselectivity. Here, we review the most representative reactions catalyzed both *in vivo* and *in vitro* by prototype flavoprotein monooxygenases, highlighting the strategies employed to produce them recombinantly, to enhance the yield of soluble proteins, and to improve cofactor regeneration in order to obtain versatile biocatalysts. Although we describe the most outstanding features of flavoprotein monooxygenases, we mainly focus on enzymes that were cloned, expressed and used for biocatalysis during the last years.

## Flavoprotein monooxygenases

Flavoprotein monooxygenases comprise a family of enzymes that participate in a wide variety of metabolic processes both in prokaryotic and eukaryotic cells. They are involved in pathways of degradation of aromatic compounds, polyketides biosynthesis, antibiotic resistance and, biosynthesis of compounds with relevant biological activities as cholesterol, antibiotics, and siderophores. Some of these enzymes participate in routes that allow microbial utilization of organic compounds as carbon and energy sources. Most flavoprotein monooxygenases are able to use molecular oxygen (O_2_) as oxygen donor to oxygenate an organic compound, a reaction that depends on a reduced flavin cofactor to activate O_2_ by electron donation. These enzymes are classified as external (EC 1.14.13) and internal monooxygenases (EC 1.13.12). External monooxygenases rely on reduced coenzymes in the form of NADPH or NADH as sources of reducing power for the flavin, whereas in internal monooxygenases the flavin is reduced by the substrate itself. Besides, there are flavin-dependent enzymes that are able to catalyze hydroxylations of organic compounds. In this case, the flavin is required to oxidize the substrate *via* a reaction in which the oxygen atom comes from water while O_2_ serves to recycle the flavin (van Berkel et al., 2006; Torres Pazmiño et al., 2010b). External flavoprotein monooxygenases contain non-covalently bound FAD or FMN and catalyze the NAD(P)H-dependent insertion of a single oxygen atom into an organic substrate while the second atom of oxygen is reduced to water. They are classified in six classes (A–F) according to structural- and sequence-related characteristics (van Berkel et al., 2006). Besides natural flavoprotein monooxygenases, modified flavins have been used as organocatalysts and novel artificial flavoenzymes have been generated by flavin re-design (de Gonzalo and Fraaije, 2013).

In the following sections we review the most representative reactions catalyzed by prototype flavoprotein monooxygenases, highlighting the strategies employed to produce them recombinantly. We describe the most outstanding features of bacterial flavoprotein monooxygenases, albeit we mainly focus on enzymes that were cloned, expressed and used for biocatalysis during the last years.

## Baeyer-villiger oxidations

The oxidation of ketones is known in organic chemistry as Baeyer-Villiger oxidation (Baeyer and Villiger, 1899). This reaction involves peracids or hydrogen peroxide to achieve the oxidation of ketones to esters or lactones. Chiral lactones are valuable intermediates toward the synthesis of natural products and analogs (reviewed in de Gonzalo et al., 2010). For Baeyer-Villiger oxidations, the enzyme-mediated transformation has become the preferred method due to its high enantio-, regio-, and chemoselectivity. Besides, the process takes place in environmentally friendly conditions, avoids the use of toxic reagents, and allows scale-up. The ability of some microorganisms to grow in a certain alcohol or ketone grabbed the attention to the enzymes involved in those metabolic routes and prompted the discovery of Baeyer-Villiger monooxygenases (BVMOs). An increasing number of BVMOs have been identified, cloned, recombinantly expressed, engineered and used for biocatalysis. This topic has been the matter of very comprehensive revisions during the last 5 years (de Gonzalo et al., 2010; Torres Pazmiño et al., 2010a; Leisch et al., 2011; Balke et al., 2012), hence the present section focuses mainly on the strategies used to overcome gene expression problems and cofactor regeneration limitations, scale-up and a summary of the most recent applications.

**Figure 1:**
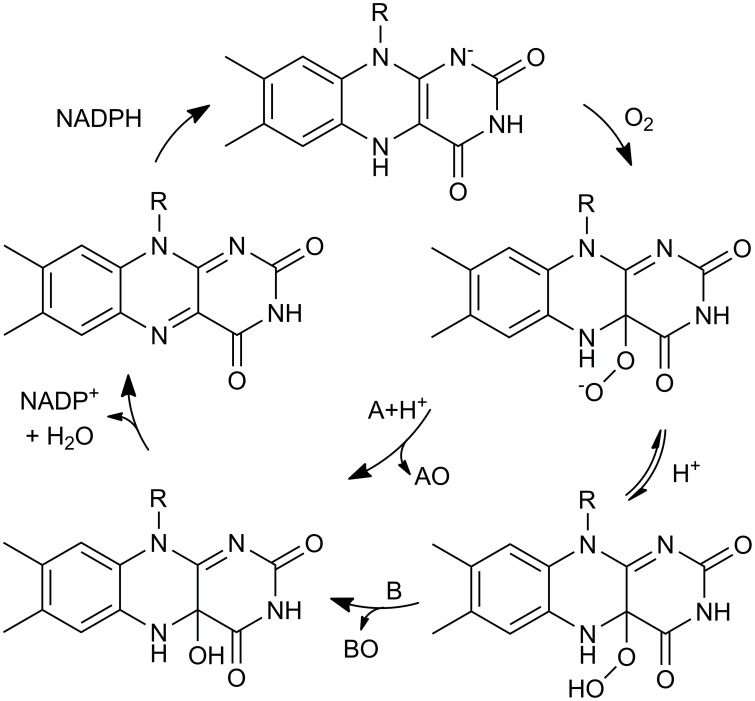
**Catalytic mechanism of Type I Baeyer-Villiger monooxygenases**.

Different types of BVMOs exist. Type I BVMOs contain FAD, depend on NADPH for catalysis and belong to the class B of flavoprotein monooxygenases while Type II BVMOs are FMN- and NADH-dependent enzymes and belong to class C of flavoprotein monooxygenases. In addition, there are atypical BVMOs that do not share their characteristics (Willetts, 1997; van Berkel et al., 2006; Torres Pazmiño et al., 2010a). Type I BVMOs are flavoenzymes that catalyze the oxidation of a linear or cyclic ketone to an ester or lactone, respectively, at the expense of molecular oxygen and NADPH. For these enzymes NADPH is the required electron donor. As a result, an oxygen atom is inserted into a carbon-carbon bond adjacent to a carbonyl group in the substrate and the other one is reduced to water. The mechanism of this reaction is proposed to proceed *via* formation and stabilization of a covalent bond between oxygen and the C4a of the isoalloxazine ring of reduced FAD. This C4a-peroxyflavin performs a nucleophilic attack on the carbonyl group of the substrate giving rise to a Criegee intermediate that rearranges spontaneously to the product (Figure 1). BVMOs can also oxygenate heteroatoms probably *via* an electrophilic mechanism (reviewed in Mihovilovic, 2006; van Berkel et al., 2006; Torres Pazmiño et al., 2008a).

In 2002, a consensus motif for Type I BVMOs was described (Fraaije et al., 2002) that was very useful for the identification of novel Type I BVMOs by genome mining (Torres Pazmiño et al., 2010). Recently, a more specific sequence motif was identified that allowed a better distinction between typical Type I BVMOs and flavin-containing monooxygenases (FMOs) (Riebel et al., 2012). More than fifty Type I BVMO genes are currently available for recombinant expression. Most of them are of bacterial origin but the cloning of the coding sequences of few eukaryotic Type I BVMOs has been reported during the last 2 years (Leipold et al., 2012; Beneventi et al., 2013; Mascotti et al., 2013).

The cyclohexanone monooxygenase from *Acinetobacter calcoaceticus* NCIMB 9871 (CHMO*_Acineto_*) is a model Type I BVMO that has been studied in-depth. It showed to be a robust biocatalyst, as expressed in *E. coli* from a pET-22b derived vector, able to catalyze selective oxygenation of a broad variety of ketones in desymmetrizations reactions, regiodivergent oxidations and kinetic resolutions (reviewed in Mihovilovic, 2006; Leisch et al., 2011). Recently, the CHMO from *Rhodococcus* sp. HI-31 was crystallized with its substrate, cyclohexanone, and with NADP^+^ and FAD at 2.4 Å resolution (Yachnin et al., 2012). A benchmark reaction catalyzed by *E. coli* cells overexpressing CHMO_*Acineto*_ was the asymmetric oxidation of the racemic bicyclo[3.2.0]hept-2-en-6-one in large scale. For this purpose, the strategies for optimization of the bioconversion included a fed-batch biotransformation, the use of resin-based *in situ* substrate feeding and product removal (SFPR) technology, a fine control of the bioprocess and a proper aeration. This bioconversion was scaled-up to pilot-plant scale (200 L) and 4.5 g/L of lactone were produced (Baldwin et al., 2008). The SFPR methodology allows the use of substrate concentrations beyond toxicity levels and avoids inhibition of the reaction by the product or substrate as their concentrations in the culture remains below inhibitory levels. Scale-up methodologies for Baeyer-Villiger biooxidation of ketones were reviewed in de Gonzalo et al. (2010). An innovative monitoring system was developed based on the use of flow-calorimetry to measure temperature changes due to Baeyer–Villiger oxygenations catalyzed by encapsulated *E. coli* expressing CHMO_*Acineto*_ (Bučko et al., 2011). Variants of CHMO_*Acineto*_ with enhanced oxidative and thermal stabilities were obtained by rational and combinational mutagenesis at M and C residues without affecting the activity or selectivity of the enzyme (Opperman and Reetz, 2010). In addition, wild-type and mutant CHMO_*Acineto*_ catalyzed the conversion of 4-ethylidenecyclohexanone into *E-* and *Z-*configured lactones, respectively, and successive reactions catalyzed by transition metals were used to produce different trisubstituted *E-* or *Z-*olefins (Zhang et al., 2013). *E. coli* cells expressing the CHMO from *Xanthobacter* sp. ZL5 (CHMO_*Xantho*_) have a very broad substrate acceptance profile and the ability to convert some bulky ketones not accepted by other BVMOs (Rial et al., 2008a, b). More recently, Alexander et al. (2012) reported the cloning and evaluation of a CHMO from the xenobiotic-degrading *Polaromonas* sp. JS666. Initial oxidation assays showed no results due to formation of inclusion bodies but upon optimization, a detailed screening of the biocatalyst could be performed (Table 1).

**Table 1:** **Baeyer-Villiger oxidation**.

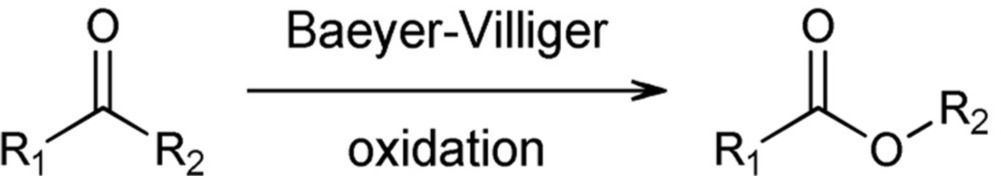			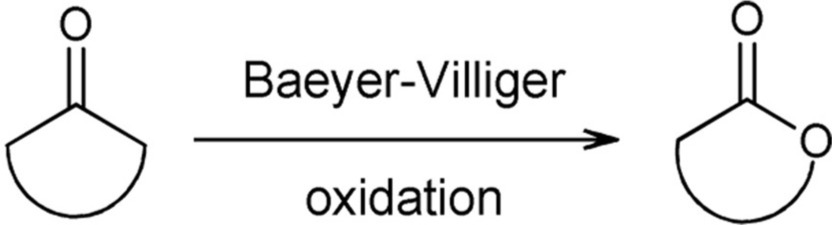
**Enzyme**	**Gene**	**Expression system**	**References^a^**
CHMO	*chmo* from *Polaromonas* sp. JS666	*E. coli* BL21 Star (DE3)/pET101/D-TOPO vector	Alexander et al., 2012
	mutants of *chnB* from *A. calcoaceticus* NCIMB 9871	*E. coli* BL21-Gold (DE3)/pET22b	Opperman and Reetz, 2010
	*chnB* from *A. calcoaceticus* NCIMB 9871 and *pos5* (NADH kinase) from *S. cerevisiae* CEN.PK2-1D	*E. coli* BL21(DE3)/pET22b (*chnB*) and ACYCDuet-1 (*pos5*)	Lee et al., 2013
	*chnB* from *A. calcoaceticus* NCIMB 9871 and *gapB* (NADP^+^-dependent GAPDH) from *B. subtilis*	*E. coli* (*gapA* null, NAD^+^-dependent glyceraldehyde-3-phosphate dehydrogenase mutant)/pET22b (*chnB*) and pDHC29 (*gapB*)	Wang et al., 2013
PAMO	different *pamO* mutants from *T. fusca*	*E. coli* TOP10/pBAD/myc-HisA	Wu et al., 2010
	different mutant *pamO* from *T. fusca*	*E. coli* TOP10/pBAD/myc-HisA	Dudek et al., 2011
	wild-type and mutant *pamO* from *T. fusca*	*E. coli* TOP10/pBAD-Tat-AldO (periplasmic expression)	Dudek et al., 2013a, b
BVMO	22 *bvmo* genes from *R. jostii* RHA1 (16 BVMOs with demonstrated activity)	*E. coli* TOP10/pBADN and pCRE2 (with phosphite dehydrogenase gene)	Riebel et al., 2012
	*bvmo* from *A*. *radioresistens* S13	*E. coli* BL21(DE3)/pT7 expression vector	Minerdi et al., 2012
STMO	wild-type and mutant *stmo* from *R. rhodochrous*	*E. coli* BL21(DE3) CodonPlus/Champion pET-vector	Franceschini et al., 2012
	wild-type *stmo* from *R. rhodochrous*	*E. coli* BL21(DE3)/pET28b	Leipold et al., 2013
SAPMO	SAPMO from *C. testosteroni* KF1	*E. coli* BL21 Star (DE3)/pET101/D-TOPO vector	Weiss et al., 2013
OTEMO	OTEMO gene from *P. putida* NCIMB 10007	*E. coli* BL21(DE3)/pET28b	Kadow et al., 2012
	OTEMO gene from *P. putida* ATCC 17453	*E. coli* BL21(DE3)/pSD80	Leisch et al., 2012
2,5-DKCMO	2,5-DKCMO gene from *P. putida* NCIMB 10007	*E. coli* BL21(DE3)/pET28b	Kadow et al., 2011, 2012
3,6-DKCMO	3,6-DKCMO gene from *P. putida* NCIMB 10007	*E. coli* BL21(DE3)/pET28b (coexpression of GroES-GroEL chaperones)	Kadow et al., 2012
2,5- and 3,6-DKCMO	2,5- and 3,6-DKCMO genes from *P. putida* NCIMB 10007 and Fre gene from *E. coli*	*E. coli* BL21(DE3)/pET28b (DKCMOs, coexpression of GroES-GroEL); pGas (*Fre* or fusion genes, L-rhamnose inducible)	Kadow et al., 2013
BVMO type II	*camE*_25−1_, *camE*_25−2_, *camE*_36_ and Fred gene from *P. putida* ATCC 17453	*E. coli* BL21(DE3)/pSD80 (each *camE* gene and tandem clones); *E. coli* BL21(DE3) pLysS/pET17b (Fred gene)	Iwaki et al., 2013
FMO (type II)	*fmo* from *S. maltophilia*	*E. coli* BL21(DE3)/pET-YSBL-LIC-3C	Jensen et al., 2012
	several *fmo* genes from *R. jostii* RHA1	*E. coli*/pBADN and pCRE2	Riebel et al., 2013a, b

a *The information shown corresponds to reports as of 2010*.

*Abbreviations: 3,6-DKCMO, 3,6-diketocamphane 1,6-monooxygenase; 2,5-DKCMO, 2,5-diketocamphane 1,2-monooxygenase; ACMO, acetone monooxygenase; BVMO, Baeyer-Villiger monooxygenase; CDMO, cyclododecanone monooxygenase; CHMO, cyclohexanone monooxygenase; CPMO, cyclopentanone monooxygenase; FMO, flavin-containing monooxygenase; OT, 2-oxo-Δ^3^-4,5,5-trimethylcyclopentenylacetic acid; OTEMO, 2-oxo-Δ^3^-4,5,5-trimethylcyclopentenylacetyl-CoA monooxygenase; PAMO, phenylacetone monooxygenase; SAPMO, 4-sulfophenyl acetate monooxygenase; STMO, steroid monooxygenase*.

The cyclopentanone monooxygenase from *Comamonas* sp. NCIMB 9872 (CPMO) is another cycloketone-converting BVMO, which can display enantiodivergent transformations with respect to the CHMO group. *E. coli* cells overexpressing the gene coding for CPMO were used as biocatalysts to oxidize an *oxo*-bridged ketone in order to obtain a heterobicyclic lactone, a key intermediate in formal total syntheses of various natural products containing a tetrahydrofuran structural motif such as *trans*-kumausyne, goniofufurone analogs and showdomycin (Mihovilovic et al., 2006). In this work, the biotransformation was carried out in a bioreactor using the *in situ* SFPR technology and the desired lactone was obtained in 70 % isolated yield (Mihovilovic et al., 2006). In a recent report, CHMO_*Xantho*_- and CPMO-mediated biooxidations of a bridged-bicyclic ketone were performed in shake-flasks scale and allowed access to both antipodal lactones in very good yields and high enantiomeric excess (e.e.). These chiral lactones were key intermediates toward (+) and (−) non-natural carba-*C*-nucleosides in high optical purity (Bianchi et al., 2013).

Other remarkable Type I BVMOs is the phenylacetone monooxygenase from *Thermobifida fusca* (PAMO) (Table 1). Its coding sequence was cloned and expressed in *E. coli* from a pBAD/myc-HisA-derived vector (Fraaije et al., 2005). PAMO can tolerate high temperatures and organic solvents (Fraaije et al., 2005; de Gonzalo et al., 2006a). The enzyme was purified, characterized and it was the first BVMO for which the three-dimensional structure was elucidated by X-ray diffraction (Malito et al., 2004). Some years afterward, Orru et al. (2011) solved the crystal structure of reduced and oxidized PAMO in complex with NADP^+^. Since the substrate profile of wild-type PAMO is mainly limited to some aromatic ketones and sulfides (de Gonzalo et al., 2005b; Rodríguez et al., 2007; Zambianchi et al., 2007), protein engineering strategies were undertaken aiming at expanding the substrate profile of PAMO without affecting its stability (Bocola et al., 2005). By a site-directed mutagenesis approach it was possible to expand the substrate range of the enzyme to some prochiral cyclic ketones, sulfides, and amines (Torres Pazmiño et al., 2007). Thermostable PAMO mutants with high activity and enantioselectivity for the conversion of 2-substituted cyclohexanones derivatives were produced by saturation mutagenesis focused on specific sites of PAMO (Reetz and Wu, 2009; Wu et al., 2010). Directed evolution and rational re-design of PAMO and other BVMOs were thoroughly reviewed recently (Zhang et al., 2012).

It has been reported that the addition of organic co-solvents to biotransformations can influence conversion and selectivity of reactions catalyzed by wild-type PAMO and variants (Rioz-Martínez et al., 2008, -Martínez et al., -Martínez et al.). Recently, de Gonzalo et al. (2012) improved the biocatalytic performance of a PAMO mutant (the variant with M446 replaced by G) in hydrophilic organic solvents and, including a weak anion exchange resin, they were able to attain the dynamic kinetic resolution of a range of benzylketones. Further optimization of PAMO biooxidations considered the buffer and ionic strength of the reaction media as well as the coupled reaction for cofactor regeneration (Rodríguez et al., 2012). To improve the biocatalyst performance, fundamental aspects of protein expression such as host strain, inducer concentration, temperature and length of induction as well as riboflavin addition were considered (van Bloois et al., 2012). This approach also evaluated biotransformation conditions including external sugars as sources of reducing power for NADPH regeneration, substrate concentration and, biotransformation temperature and length. Recently, Dudek et al. (2013b) developed a screening method based on periplasmically expressed PAMO aiming at enabling complete access of substrates to the enzyme and facilitating NADPH recycling by externally added phosphite dehydrogenase (PTDH) from *Pseudomonas stutzeri* WM88. The *pamO* gene was cloned into a pBAD-derived plasmid between an N-terminal Tat-dependent signal sequence of the endogenous *E. coli* protein TorA and a C-terminal Myc epitope/His-tag. The Tat-PAMO protein was functionally expressed in the periplasm of *E. coli* cells and this system was used together with the PTDH-based regeneration system for biotransformations. Just recently, this analysis was extended to the screening of a library of PAMO mutants, which resulted in the isolation of a quadruple mutant with the same thermostability as the wild-type enzyme but with an extended substrate scope (Dudek et al., 2013a) (Table 1).

Another available BVMO is the cyclopentadecanone monooxygenase from *Pseudomonas* sp. HI-70 (CPDMO). Its gene was cloned in 2006 and initial assays detected activity toward large ring ketones (C11-C13), substituted cyclohexanones (Iwaki et al., 2006) and ketosteroids (Beneventi et al., 2009). The biocatalytic performance of CPDMO was evaluated extensively in 2011 and showed a behavior similar to CHMO within desymmetrizations and kinetic resolutions, but performed particularly interesting in regiodivergent oxidations (Fink et al., 2011). Another robust biocatalyst is cyclododecanone monooxygenase from *Rhodococcus ruber* SC1 (CDMO), which was used as a case study to show the potentials of a new tool for chiral catalysts assessment (Fink et al., 2012). The 4-hydroxyacetophenone monooxygenase (HAPMO) from *Pseudomonas fluorescens* ACB has been available for many years (Kamerbeek et al., 2001). In 2009, the gene encoding for a HAPMO from *Pseudomonas putida* JD1 was cloned, functionally expressed and characterized (Rehdorf et al., 2009). Soluble protein production was problematic thus several strategies were undertaken to circumvent this limitation. Expression of the HAPMO-encoding gene was assayed from two different plasmids and in several bacterial hosts, in media with different composition, at various temperatures, in the presence or absence of FMN and by co-expression with molecular chaperones. By biotransformations in crude cell extracts it was found that this enzyme preferentially oxidizes aryl-aliphatic ketones (Rehdorf et al., 2009). In a following work, the biooxidation of the aromatic ketone 3-phenyl-2-butanone was scaled-up in a bioreactor and yields improved by the use of adsorbent resins for an *in situ* SFPR (Geitner et al., 2010).

Other BVMOs have been newly reported (Table 1). A set of predicted 22 *bvmo*-encoding genes from *Rhodococcus jostii* RHA1 were cloned but only 12 of them could be expressed as soluble active enzymes (Szolkowy et al., 2009). However, by applying a high-throughput cloning strategy and optimized expression conditions Riebel et al. (2012) were able to express the 22 probable *bvmo* genes identified in the genome of *R. jostii* RHA1 in soluble form. They cloned the selected genes under the control of *araBAD* promoter directly or as a fusion with the PTDH gene (Riebel et al., 2012). Other recently expressed *bvmo* genes include the *almA* gene from *Acinetobacter radioresistens* S13 that encodes a BVMO involved in the subterminal oxidation of alkanes (Minerdi et al., 2012), a steroid monooxygenase (STMO) from *Rhodococcus rhodochrous* which crystal structure (Franceschini et al., 2012) and substrate profile (Leipold et al., 2013) were determined, a 4-sulfoacetophenone monooxygenase (SAPMO) from *Comamonas testosteroni* KF-1 that is involved in the biodegradation of 4-sulfophenylcarboxylates (Weiss et al., 2013), and the 2-oxo-Δ3-4,5,5-trimethylcyclopentenylacetyl-CoA monooxygenase (OTEMO) from *P. putida* NCIMB 10007 (Kadow et al., 2012) and from *P. putida* ATCC 17453 (Leisch et al., 2012). This enzyme participates in the degradation of camphor in the native microbial host but, recombinantly expressed in *E. coli*, it is able to accept α,β-unsaturated monocyclic and bicyclic ketones (Kadow et al., 2012).

The strict dependence of BVMOs on NADPH for catalysis certainly impairs the practical applications of these enzymes due to the high costs of NADPH or to the requirement of a cofactor regeneration system. The possibility to carry out the desired biotransformation in whole-cell systems is a beneficial alternative since the cell itself provides the NADPH. The co-expression of glucose-6-phosphate dehydrogenase or the addition of carbohydrates to the culture media can improve NADPH regeneration by the host cells (Walton and Stewart, 2002; Lee et al., 2007). Besides, several other options are available to regenerate NADPH for BVMO activity (recently reviewed in de Gonzalo et al., 2010). Some coenzyme regeneration systems are based on a coupled enzymatic reaction that produces NADPH at the expense of an auxiliary substrate. Typical pure enzymes used for the regeneration of NADPH include glucose-6-phosphate dehydrogenase, PTDH, alcohol dehydrogenase and glucose dehydrogenase. However, these systems need to be added to the activity assays. In the last years, an alternative strategy was developed in which fusion proteins between a PTDH and certain BVMOs were produced and evaluated as self-sufficient biocatalysts (Torres Pazmiño et al., 2008b; 2009). Other approaches based on the chemical (de Gonzalo et al., 2005a) or photochemical (Hollmann et al., 2007) regeneration of the flavin bound to the BVMO have also been investigated. Most recently, two strategies to improve NADPH regeneration were presented and tested in BVMO-mediated biotransformations (Table 1). In one approach, a NADH kinase from yeast was used for the direct phosphorylation of NADH to NADPH in *E. coli* cells producing CHMO_*Acineto*_ (Lee et al., 2013). This approach enhanced the oxidation of cyclohexanone in a fed-batch biotransformation and doubled the productivity of ε-caprolactone when compared with the control lacking the NADH kinase (Lee et al., 2013). The other approach proposed a strategy to increase NADPH bioavailability by replacing the native NAD^+^-dependent glyceraldehyde-3-phosphate dehydrogenase *gapA* gene in *E. coli* with a NADP^+^-dependent *gapB* gene from *Bacillus subtilis*, hence producing in *E. coli* a NADP^+^-dependent glyceraldehyde-3-phosphate dehydrogenase from a plasmid and the CHMO_*Acineto*_ from a compatible expression vector (Wang et al., 2013).

Two additional BVMOs (named 2,5-diketocamphane 1,2-monooxygenase (2,5-DKCMO) and 3,6-diketocamphane 1,6-monooxygenase (3,6-DKCMO)) that participate in the camphor-degrading metabolic route in *P. putida* NCIMB 10007 are Type II BVMOs (Kadow et al., 2011, 2012). They are two-component systems consisting of a monooxygenase and a reductase, and depend on FMN- and NADH for activity. The genes encoding the monooxygenase subunit of 2,5-DKCMO and 3,6-DKCMO were recombinantly expressed in *E. coli* (Kadow et al., 2011, 2012). The expression of the gene encoding the oxygenase subunit of 3,6-DKCMO required the assistance of molecular chaperones for enhanced soluble expression (Kadow et al., 2012) (Table 1). These biocatalysts were able to convert mainly bicyclic ketones. Three *camE* genes from *P. putida* ATCC 17453 coding for different monooxygenase subunits of DKCMO isoenzymes were cloned and expressed (Iwaki et al., 2013). In addition, one FMN reductase (*Fred*) gene from the same bacteria was identified and cloned individually or in tandem with the respective 2,5-, or 3,6-DKCMO-coding genes. Pairs DKCMO-Fred were able to convert bicyclic ketones with enantiomeric specificity in recombinant whole-cell systems (Iwaki et al., 2013) (Table 1). Recently, a flavin-reductase Fre from *E. coli* was reported as an appropriate partner for providing reduced FMN to either 2,5- or 3,6-DKCMO from *P. putida* NCIMB 10007. Couples DKCMO-Fre were able to oxidize camphor and norcamphor in the presence of NADH generated by formate dehydrogenase (FDH) from *Candida boidinii* (Kadow et al., 2013).

Besides BVMOs, FMOs are capable of catalyzing Baeyer-Villiger oxidations (Table 1). Jensen et al. (2012) reported the ability of an FMO from *Stenotrophomonas maltophilia* (SMFMO) to catalyze some Baeyer-Villiger oxidations as well as sulfoxidations and to use both NADH and NADPH. The codon-optimized synthetic gene was cloned and SMFMO was produced in *E. coli*, purified and its crystal structure elucidated. Just recently, Riebel et al. (2013a) cloned, expressed in *E. coli* and explored the catalytic potential of several novel flavoprotein monooxygenases from *R. jostii* RHA1 with homology to FMOs. The authors studied the ability of the novel enzymes, classified as Type II FMOs, to convert phenylacetone, (±)-bicyclo[3.2.0]hept-2-en-6-one and methyl phenyl sulfide (thioanisole). These results were further extended by in-depth screening in whole-cell systems (Riebel et al., 2013b).

## Epoxidations

Epoxides are valuable precursors for synthetic applications toward bioactive compounds, thus in this section we describe attractive epoxidations carried out by recombinant flavoprotein monooxygenases.

Two-component styrene monooxygenases belong to the class E of flavoprotein monooxygenases (Table 2). The first step in the metabolic utilization of styrene in *Pseudomonas* sp. VLB120 is catalyzed by an oxygenase (StyA) and a NADH-flavin oxidoreductase (StyB). The genes coding for this enzyme (StyAB) were identified, cloned and expressed in *E. coli* (Panke et al., 1998). In order to investigate the relationships between styrene epoxidation, StyAB production, cell growth and carbon metabolism, two-liquid-phase continuous cultures of *E. coli* expressing *styAB* genes of *Pseudomonas* sp. VLB120 were performed in a 3 L-stirred reactor (Bühler et al., 2008). The two-phase system made possible the operation of the biocatalyst at subtoxic non-inhibitory substrate and product concentrations. It also allowed control of the epoxidation rate by varying styrene feed concentration (Bühler et al., 2008). In order to improve styrene biotransformation by *E. coli* expressing the two-component styrene monooxygenase from *P. putida* CA-3, the corresponding coding gene was subjected to *in vitro* evolution followed by an indole bioconversion-based screening (Gursky et al., 2010). Ukaegbu et al. (2010) reported the X-ray crystal structure of the N-terminally His-tagged oxygenase subunit of the styrene monooxygenase from *P. putida* S12. Based on this structural data and aiming at improving the preference of the enzyme toward α-substituted styrene, point mutations were introduced in the styrene monooxygenase from *Pseudomonas* sp. LQ26 (Lin et al., 2010, 2011a, b) by site-directed mutagenesis (Qaed et al., 2011). This procedure allowed the development of mutants with increased reactivity toward α-substituted styrene derivatives. By a similar rational design approach, a different set of mutations was found to exhibit increased epoxidation activity toward styrene and *trans*-β-methyl styrene compared with the wild-type enzyme. Interestingly, one of these mutants showed reversed enantiomeric preference toward 1-phenylcyclohexene (Lin et al., 2012) (Table 2).

**Table 2:** **Epoxidation**.

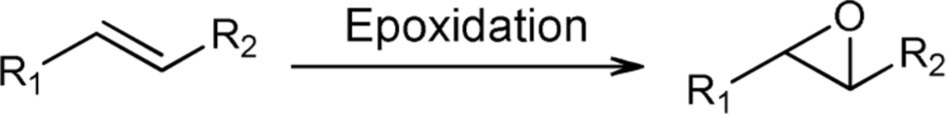			
**Enzyme**	**Gene**	**Expression system**	**References^a^**
SMO	wild-type and mutant *styAB* fragment from *P. putida* CA-3	*E. coli* XL-10 Gold/pBluescript II KS; *E. coli* BL21(DE3)/pRSET-B	Gursky et al., 2010
	wild-type and several mutant *styAB2* from *Pseudomonas* sp. LQ26	*E. coli* BL21(DE3)/pET28a	Lin et al., 2010, 2011a, 2012
	different mutant *styAB2* from *Pseudomonas* sp. LQ26	*E. coli* BL21(DE3)/pET28a	Qaed et al., 2011
	*styA1/StyA2B* from *R. opacus* 1CP	*E. coli* BL21(DE3) pLysS/pET16bP	Tischler et al., 2009, 2010

a *The information shown mainly corresponds to reports as of 2010*.

*Abbreviation: SMO, styrene monooxygenase*.

van Hellemond et al. (2007) reported the discovery of a novel styrene monooxygenase (SmoA) in a metagenomic library derived from loam soil. *In vitro* activity assays using crude cell extracts of bacteria producing SmoA evidenced epoxidation of styrene and styrene derivatives to the corresponding (*S*)-epoxides with excellent e.e. In *Rhodococcus opacus* 1CP, a self-sufficient styrene monooxygenase was reported that harbors in the same polypeptide chain a monooxygenase and a NADH-flavin oxidoreductase (StyA2B) (Tischler et al., 2009). Moreover, a multifunctional monooxygenase system (StyA1/StyA2B) was recently described in the same microorganism as composed of a single styrene monooxygenase (StyA1) and the StyA2B polypeptide (Tischler et al., 2010) (Table 2). Purified StyA2B was able to oxidize styrene, 2-chlorostyrene, 3-chlorostyrene, 4-chlorostyrene, 4-methylstyrene, and dihydronaphthalene.

Surprisingly, the epoxidation of an electron-rich C=C functionality in the *oxo*-bridged bicyclic ketone (1R,5S)-8-oxabicyclo[3.2.1]oct-6-en-3-one was catalyzed by CHMO_*Xantho*_, representing the first report of a BVMO involved in this conversion (Rial et al., 2008a).

## Sulfoxidations

Enantioselective sulfoxidations are difficult to accomplish chemically and, therefore enzyme-mediated sulfoxidations have attracted the attention of chemists and biochemists during the last decades.

In 2007, it was shown that crude cell extracts of bacteria producing SmoA can catalyze sulfoxidation reactions with high enantioselectivity toward aromatic sulfides (van Hellemond et al., 2007). The multifunctional monooxygenase system StyA1/StyA2B from *R. opacus* 1CP (Tischler et al., 2009, 2010) is also capable of oxidizing thioanisole (Table 3). In addition, oxidation of sulfides can be readily catalyzed by *E. coli* cells expressing the two-component NADH-dependent styrene monooxygenase from *P. putida* CA-3 (Nikodinovic-Runic et al., 2013). Boyd et al. (2012) have demonstrated that this biocatalyst is able to *S*-oxidize benzo[b]thiophene and, other nine sulfur-containing compounds, including thioanisole and some substituted analogs, benzo[b]thiophene, and 2-methylbenzo[b]thiophene were accepted as substrates as well (Nikodinovic-Runic et al., 2013). The enzyme that had been previously engineered for improved alkene epoxidation (Gursky et al., 2010) showed an increased *S*-oxidation capability when compared with the wild-type form, being the sulfur atom in the thiophene ring a better target than the sulfur atom in an alkyl chain (Nikodinovic-Runic et al., 2013).

**Table 3:** **S-oxidation**.

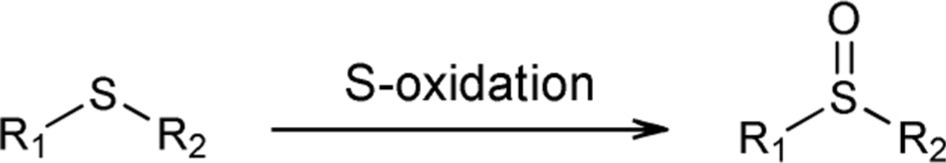			
**Enzyme**	**Gene**	**Expression system**	**References^a^**
SMO	*styA1/StyA2B* from *R. opacus* 1CP	*E. coli* BL21(DE3) pLysS/pET16bP	Tischler et al., 2009, 2010
	wild-type and mutant *styAB* fragment from *P. putida* CA-3	*E. coli* BL21(DE3)/pRSET-B	Gursky et al., 2010; Boyd et al., 2012; Nikodinovic-Runic et al., 2013
PAMO	different mutant *pamO* from *T. fusca*	*E. coli* TOP10/pBAD/myc-HisA	Dudek et al., 2011
	wild-type and mutant *pamO* from *T. fusca*	*E. coli* TOP10/pBAD-Tat-AldO plasmid (periplasmic expression)	Dudek et al., 2013a, b
BVMO	9 *bvmo* genes from *R. jostii* RHA1	*E. coli* TOP10/pBADN and pCRE2 (phosphite dehydrogenase gene)	Riebel et al., 2012
	*bvmo* gene from *A. radioresistens* S13	*E. coli* BL21(DE3)/pT7 expression vector	Minerdi et al., 2012
FMO (type I)	*fmo* from *Methylophaga* sp. SK1	*E. coli* TOP10/pCRE2 (soluble NADPH regenerating phosphite dehydrogenase)	o, Gotor and Fraaije, Rioz-Martínez et al.
	*tmm* gene from *M. silvestris*	*E. coli* BLR(DE3) pLysS/pET28a	Chen et al., 2011
FMO (type II)	*fmo* gene from *S. maltophilia*	*E. coli* BL21(DE3)/pET-YSBL-LIC-3C	Jensen et al., 2012
	several *fmo* genes from *R. jostii* RHA1	*E. coli*/pBADN and pCRE2	Riebel et al., 2013a
CHMO	*chmo* gene from *A. calcoaceticus* NCIMB 9871 and mutant *fdh* (formate dehydrogenase) gene from *C. boidinii*	*E. coli* BL21(DE3)/pET22b (*chmo*) and pACYCDuet-1 (*fdh* or both genes in tandem)	Zhai et al., 2013

a *The information shown mainly corresponds to reports as of 2010*.

*Abbreviations: BVMO, Baeyer-Villiger monooxygenase; FMO, flavin-containing monooxygenase; CHMO, cyclohexanone monooxygenase; fdh, formate dehydrogenase; HAPMO, 4-hydroxyacetophenone monooxygenase; PAMO, phenylacetone monooxygenase; SMO, styrene monooxygenase; tmm, trimethylamine monooxygenase*.

The BVMOs are also capable of stereoselective sulfoxidations. In 2005, the ability of PAMO to oxidize aromatic sulfides was proved but the sulfoxides displayed poor e.e. (de Gonzalo et al., 2005b; Fraaije et al., 2005). The enzymatic oxidation of sulfides mediated by pure PAMO from *T. fusca*, HAPMO from *P. fluorescens* ACB and ethionamide monooxygenase (EtaA) from *Mycobacterium tuberculosis* was evaluated in several aqueous-organic media (de Gonzalo et al., 2006a). More recently, the HAPMO from *P. putida* JD1 was challenged in the sulfoxidation of methyl-4-tolyl sulfide using crude cell extracts (Rehdorf et al., 2009) and, the repertoire of chiral sulfoxides accessed was extended by using both PAMO and HAPMO from *P. fluorescens* ACB as crude cell-free extracts (Rioz-Martínez et al., 2010a). The stereoselectivity of PAMO-mediated oxidation of several prochiral thio-ethers has been enhanced by mutagenesis of M446 to G (Torres Pazmiño et al., 2007) and solvent engineering methodologies were explored in order to expand the applications of wild-type and M446G PAMO (de Gonzalo et al., 2012). The oxidation of benzyl methyl sulfide was evaluated in seventeen combinations of buffer/co-solvent and compared with the reaction in aqueous medium. The reaction in Tris-HCl pH 9.0 containing 5 % methanol rendered the corresponding sulfoxide in high conversion and good e.e. with only low levels of sulfone as by-product (de Gonzalo et al., 2012). Besides, Rodríguez et al. (2012) analyzed the effect of several enzymatic cofactor regeneration systems and cofactor concentrations in the oxidation of thioanisole by PAMO. By protein engineering, amino acidic positions were identified in PAMO that alter conversion and selectivity of *S*-oxidations (Dudek et al., 2011). In this investigation, the bulky prochiral benzyl phenyl sulfide, which is only very poor substrate for wild-type PAMO, was readily oxidized by the M446G mutant (Dudek et al., 2011). Therefore, it was selected as substrate to evaluate periplasmic expression of PAMO variants in order to establish a whole-cell screening method for the assessment of libraries of PAMO toward the identification of mutants with altered biocatalytic performances (Dudek et al., 2013a, b).

Nine BVMOs from *R. jostii* RHA1 that were cloned and expressed by Riebel et al. (2012) showed confirmed *S*-oxidation activity on thioanisole, benzyl phenyl sulfide, benzyl ethyl sulfide or ethionamide. Besides, the BVMO coded by the gene *almA* from *A. radioresistens* S13 is also able to act on ethionamide to give the corresponding *S*-oxide (Minerdi et al., 2012) (Table 3). Most recently, the oxidation of thioanisole by CHMO_*Acineto*_ was investigated using FDH as NADPH recycling system (Zhai et al., 2013). The authors constructed a whole-cell biocatalyst able to co-express the *chmo_*Acineto*_* and a modified *fdh* gene from *C. boidinii* that can utilize NADP^+^ efficiently. Several conditions including concentration of the biocatalyst and substrate, pH, temperature and time of reaction as well as addition of dimethylsulfoxide and NADP^+^ were evaluated to optimize the biocatalytic reaction (Zhai et al., 2013).

The mFMO from *Methylophaga* sp. SK1 was the first bacterial Type I FMO reported in the literature (Choi et al., 2003). The authors cloned, expressed and characterized the recombinant enzyme. They determined its activity on *N*- and *S*-containing compounds such as trimethylamine and thiourea and emphasized its ability to produce indigo blue. Alfieri et al. (2008) noted an unexpected activity of this enzyme on dimethylsulfoxide. Few years later, Rioz-Martínez et al. (2011) reported the fusion of an optimized and thermostable PTDH with the mFMO from *Methylophaga* sp. SK1 and evaluated its ability to act on several prochiral sulfides using NADPH as electron donor. Amongst the accepted sulfides, thioanisole was the best substrate but the chiral sulfoxides were obtained in moderate e.e. Some substituted thioanisole derivatives as well as other (hetero)aromatic sulfides and alkyl butyl sulfides were also oxidized to the corresponding sulfoxides, showing moderate to very good enantioselectivity (Rioz-Martínez et al., 2011). Another bacterial FMO, the trimethylamine monooxygenase TMM from *Methylocella silvestris*, was cloned, functionally expressed in *E. coli* and evaluated in the oxidation of dimethylsulfide and dimethylsulfoxide (Chen et al., 2011).

The recently described Type II FMOs are also able to oxidize sulfides, as it was shown for the SMFMO from *S. maltophilia* on thioanisole, *p*-tolyl methyl sulfide, *o*- and *p*-chlorophenyl methyl sulfide, benzyl methyl sulfide, and phenyl ethyl sulfide and, for the set of *R. jostii* Type II FMOs on thioanisole mainly (Jensen et al., 2012; Riebel et al., 2013a).

It is worth noting that de Gonzalo et al. (2011) created artificial flavoenzymes that behaved as self-sufficient flavoprotein monooxygenases capable of stereocomplementary hydrogen peroxide-driven sulfoxidations by reconstitution of the apo form of a riboflavin-binding protein isolated from eggs with modified flavin derivatives.

## *N*-hydroxylations and *N*-oxidations

*N*-hydroxylating flavoprotein monooxygenases (NMOs) mediate the FAD-dependent oxidation of amines using NADPH as electron donor in the presence of molecular oxygen. Typical representatives are L-ornithine hydroxylases and L-lysine hydroxylases.

In *Pseudomonas aeruginosa*, L-ornithine hydroxylase catalyzes the hydroxylation of the side chain amine of L-ornithine to produce the corresponding hydroxylamine, the initial step in the biosynthesis of the siderophore pyoverdine. After further modifications the hydroxylamine produces a hydroxymate functional group that is able to chelate ferric ions. The gene coding for L-ornithine hydroxylase (*PvdA*) from *P. aeruginosa* PAO1 was cloned and overexpressed in *E. coli* as a His-tagged fusion (Ge and Seah, 2006; Meneely and Lamb, 2007). The authors characterized it biochemically and its specificity toward several amino acids was investigated. In 2011, two structures of this enzyme, one in its oxidized state and the other in its reduced state, were presented for the first time (Olucha et al., 2011). An L-ornithine hydroxylase was identified in the proteome of *R. jostii* RHA1 (Bosello et al., 2012) and, its coding sequence was cloned and expressed from a pBAD-based expression vector just recently (Riebel et al., 2013a).

The Type I mFMO from *Methylophaga* sp. SK1 is capable of *N*-oxygenations as it was demonstrated for trimethylamine, cysteamine, thiourea, and other *N*-containing compounds (Choi et al., 2003; Alfieri et al., 2008). This activity on trimethylamine was further explored upon fusion of mFMO with the PTDH for self-sufficient cofactor regeneration (Rioz-Martínez et al., 2011). The TMM from *M. silvestris* was also active on methylated amines such as trimethylamine and dimethylamine (Chen et al., 2011). This enzyme is proposed to catalyze the oxidation of trimethylamine to trimethylamine *N*-oxide both in eukaryotes and prokaryotes. In the same work, the authors extended the substrate analysis to four additional bacterial TMMs from *Roseovarius* sp. 217, *Ruegeria pomeroyi* DSS-3, *Pelagibacter ubique* HTCC1002, and *P. ubique* HTCC7211, which were cloned from genomic DNA or synthetic genes, recombinantly expressed and purified from *E. coli* (Chen et al., 2011).

## Hydroxylations

The 4-hydroxybenzoate hydroxylases (PHBH) are classified as class A of flavoprotein monooxygenases, they are encoded by a single gene and contain tightly bound FAD to the sole dinucleotide binding domain present in these enzymes. They depend on NADPH or NADH as electron donors for flavin reduction. Prototype enzymes are PHBH from *P. fluorescens* and*P. aeruginosa*. Physiologically, PHBH participates in routes of degradation of aromatic carbon compounds in soil bacteria by catalyzing the hydroxylation in position 3 of the activated 4-hydroxybenzoate to generate 3,4-dihydroxybenzoate (protocatechuate) that finally enters the β-ketoadipate pathway. The genes encoding the PHBH from *P. aeruginosa* and *P. fluorescens* were cloned and expressed in *E. coli* more than 20 years ago (Entsch et al., 1988; van Berkel et al., 1992). More recently, by means of a combinatorial mutagenesis approach starting from available single mutations of the PHBH from *P. fluorescens* NBRC 14160 multiple properties of the enzyme were simultaneously improved (Suemori and Iwakura, 2007). Subsequently, 53 conserved residues from 92-aligned PHBH primary sequences and 19 non-conserved but presumable functional residues from *P. fluorescens* NBRC 14160 PHBH were substituted with each of the natural amino acids and, activity as well as NADPH reaction specificity were evaluated (Suemori, 2013). Recently, the genes coding for a 4-hydroxybenzoate hydroxylase (*pobA*) and a 3-hydroxybenzoate hydroxylase (*mobA*) from the moderate halophyte *Chromohalobacter* sp. HS-2 were cloned and overexpressed in *E. coli* (Kim et al., 2012). They are part of a cluster containing the genes responsible for the metabolism of benzoate and hydroxybenzoate in this bacterium (Kim et al., 2008, 2012). Both genes were cloned into the pET-28a(+) vector in order to obtain a fusion to a carboxyl-terminal His-Tag. Initial overexpression experiments gave mostly insoluble His-tagged proteins. Therefore, the culture was subjected to heat shock to induce the expression of *E. coli* DnaK and DnaJ molecular chaperones prior to IPTG-dependent expression of the hydroxylase genes in an attempt to improve protein solubility. Bioconversion of 4- or 3-hydroxybenzoates to protocatechuate was tested in resting cells producing the recombinant 4- or 3-hydroxybenzoate hydroxylase, respectively. The authors reported an increase in product formation, reflected in enhanced bioconversion efficiency, when the reaction was carried out after heat-induction of molecular chaperones (Kim et al., 2012). In 2008, the *mobA* gene from *C. testosteroni* GZ39 coding for 3-hydroxybenzoate hydroxylase (3HB4H) was cloned and subjected to directed evolution by error-prone PCR. With only a single point mutation, the enzyme able to hydroxylate phenolic acids was transformed into an enzyme that can also act on phenol (Chang and Zylstra, 2008).

The recombinant expressions of the 3-hydroxybenzoate 6-hydroxylase (3HB6H) from *Pseudomonas alcaligenes* NCIMB 9867 P25X and from *Polaromonas naphthalenivorans* CJ2 were reported in 2005 and 2007, respectively (Gao et al., 2005; Park et al., 2007). Recently, a 3HB6H from *R. jostii* RHA1 was cloned in the pBAD/Myc-His vector, overexpressed in *E. coli* and characterized biochemically (Montersino and van Berkel, 2012). This FAD-dependent enzyme introduces the hydroxyl group in *p*-position with respect to the previous OH on a series of *o*- or *m*-substituted 3-hydroxybenzoate derivatives. In this study, the authors performed a survey for flavin-dependent hydroxylases in *R. jostii* RHA1 genome and found several hydroxylases that belong to class A of flavoprotein monooxygenases (Montersino and van Berkel, 2012). The crystal structure of the recombinant 3HB6H from *R. jostii* RHA1 was solved recently (Montersino et al., 2013).

Due to their ability to oxidize monophenol to *o*-diphenol compounds, 4-hydroxyphenylacetate 3-hydroxylases (HPAH) are attractive biocatalysts (Lee and Xun, 1998). The enzymes are two-component systems formed by reductase and hydroxylase subunits. Thotsaporn et al. (2004) cloned each subunit in pET-11 derived vectors, expressed them in *E. coli*, and purified and characterized the recombinant enzymes. Later, the *E. coli* W HPAH was cloned in a pETDuet vector, expressed in *E. coli* BL21(DE3) and the whole-cell system was used for the biotransformation of 4-substituted halophenols to the corresponding catechols in shake- flasks and in a 5 L bioreactor (Coulombel et al., 2011). Another group of two-component flavin-dependent monooxygenases that catalyze the oxygenation of 4-hydroxyphenylacetate is represented by the HPAH from *P. aeruginosa*. It was cloned, expressed in *E. coli* and characterized. It was able to oxidize tyrosol to hydroxytyrosol and various phenols (Chakraborty et al., 2010). Recently, both genes coding for HPAH from *P. aeruginosa* PAO1 were cloned into a pETDuet-1 vector and expressed in *E. coli* cells. Biotransformation of several compounds was tested in whole-cell systems and the oxidation of *p*-coumaric acid to caffeic acid was scaled-up both in the absence and in the presence of glucose or glycerol by stepwise increases in substrate concentration in order to avoid substrate inhibition of the enzymatic activity (Furuya and Kino, 2013).

## Other reactions catalyzed by flavoprotein monooxygenases

The oxidation of indole by microbial oxygenases has been studied during the last 30 years (Ensley et al., 1983). Amongst the recombinant enzymes utilized for this purpose, phenol hydroxylases and styrene monooxygenases are worth mentioning (Doukyu et al., 2003; Gursky et al., 2010). An interesting application of indole biooxidation by styrene monooxygenases was the development of a colorimetric method for the screening of a directed evolution library of *styAB* from *P. putida* CA-3 (Gursky et al., 2010).

Choi et al. (2003) showed the ability of the mFMO from *Methylophaga* sp. SK1 to produce indigo in *E. coli* and, in the presence of tryptophan, they could increase the production of indigo up to 160 mg/L. To further improve this process, the original plasmid was subjected to deletions in the upstream region of the *fmo* ORF from *Methylophaga aminisulfidivorans* MP^T^ chromosomal DNA previously cloned. The best producing strain was selected and the composition of the medium as well as pH and temperature for the production of indigo were optimized. As result, the authors could produce 920 mg/L of bio-indigo from the recombinant *E. coli* cells (Han et al., 2008). In 2011, the same research group reported the production of indigo in large scale batch fermentation and in continuous cultivation from the above-mentioned recombinant strain. The latter fermentation mode allowed them to accumulate 23 g of bio-indigo in 110 h (Han et al., 2011). The observation that cultures of cells producing the fusion PTDH-mFMO turned blue motivated an investigation of the ability of isolated PTDH-mFMO to synthesize some indigoid derivatives and the results were readily visualized as different colors of the reaction mixtures (Rioz-Martínez et al., 2011). The first oxidation of indole catalyzed by a BVMO was reported in 2007 for the M446G mutant of PAMO (Torres Pazmiño et al., 2007), when cultures expressing this recombinant enzyme turned blue due to the formation of indigo blue.

Some Type I BVMOs have also the ability to oxidize boron-containing compounds (Branchaud and Walsh, 1985; Walsh and Chen, 1988). In 2005, PAMO from *T. fusca* was shown to oxidize phenylboronic acid to phenol (de Gonzalo et al., 2005b). A year later the same research group reported a similar result for HAPMO from *P. fluorescens* ACB (de Gonzalo et al. 2006b). These studies were extended to a variety of boron-containing acetophenones, vinyl boron compounds and racemic boron-containing compounds and two other BVMOs were evaluated (i.e., the PAMO M446G mutant and CHMO_*Acineto*_) (Brondani et al., 2011). The chemoselectivity of the reactions was variable and depended on the biocatalyst, being the boron oxidation exclusively preferred over Baeyer-Villiger oxidation on 3-substituted acetophenones for wild-type and M446G PAMO. The selectivity between epoxidation and boron oxidation was investigated in vinyl boron compounds and only boron oxidation was reported in some cases. The excellent chemoselectivity of PAMO was employed to attain the kinetic resolution of boron-containing compounds giving chiral alcohols and chiral boron compounds in high e.e. (Brondani et al., 2011). In a following report, the enantioselectivity of the BVMOs for oxidative kinetic resolutions of racemic cyclopropyl boronic esters, phenylethyl boronates, and β-boronated carboxylic esters was investigated (Brondani et al., 2012a). In addition, recombinant PAMO efficiently mediated the chemoselective oxidation of some organoselenium acetophenones to the corresponding selenoxides (Andrade et al., 2011) and, a chiral selenium compound was afforded by the kinetic resolution of a racemic selenium-containing aromatic compound with high e.e. in a reaction mediated by PAMO (Brondani et al., 2012b).

## Concluding remarks

**Figure 2:**
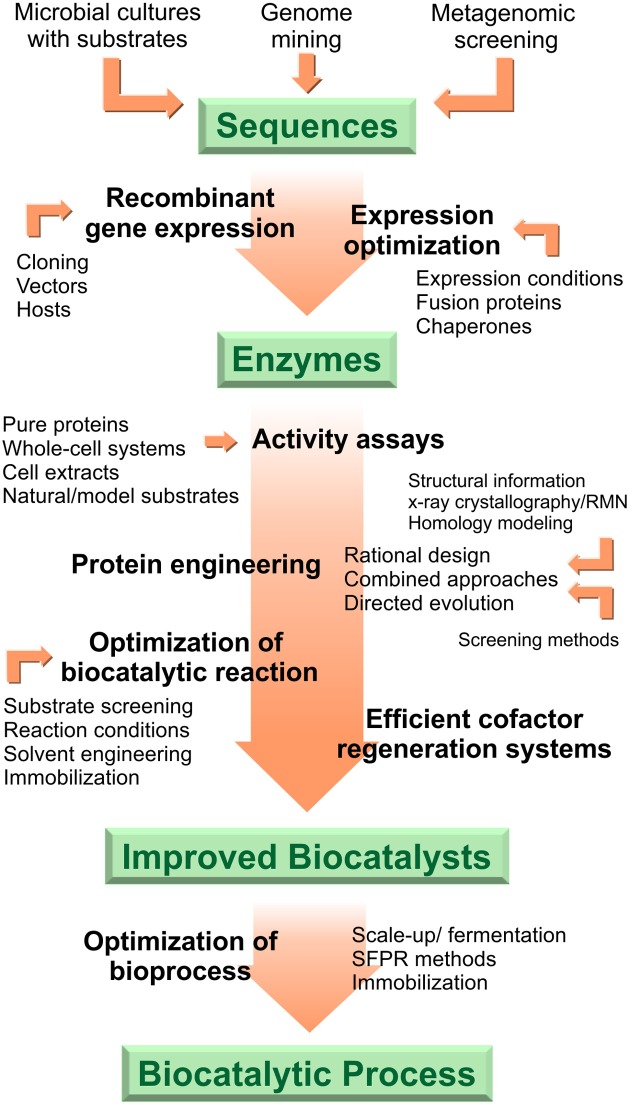
**General strategy for the development of recombinant biocatalysts**.

Biocatalysis is an environmentally friendly strategy for the elaboration of fine chemicals, natural products or other biologically active compounds. During the last decades enormous efforts have been done to satisfy the demands of biocatalysts for organic synthesis. However, multidisciplinary and coordinate work is still required to enlarge the repertoire of accessible reactions and compounds. The development of recombinant biocatalysts for organic synthesis and industrial applications involves multiple steps beginning from sequence selection up to bioprocess improvement (Figure 2). Aiming at describing and exemplifying this entire process, in the preceding sections we presented recent work and the state of the art on flavoprotein monooxygenases-mediated reactions for the creation of selective and stable biocatalysts as well as robust biotransformation processes. Bioinformatics analysis, recombinant DNA technology and protein engineering methods are part of the basic toolkit toward optimized redox biocatalysts. The sequence of interest can derived from natural or synthetic origin. Once it is cloned, selection of convenient expression vectors and improved hosts, optimization of growing and induction media or conditions and assisted protein folding can help reach proper recombinant expression levels. Considering the critical requisite of flavoprotein monooxygenases for cofactor recycling, activity of the biocatalysts can be evaluated in formats ranging from whole-cell systems to pure enzymes. Protein engineering techniques such as directed evolution, rational re-design and *de-novo* design of enzymes allow the expansion of the range of biocatalysts available and the development of tailored enzymes. Innovations on solvent or reaction medium engineering have also a huge impact on the biocatalytic outcome. Immobilization methods, strain improvement by metabolic engineering, and scale-up procedures under fine bioprocess control are further valuable tools for the development of a successful biocatalytic process for the industry.

### Conflict of interest statement

The authors declare that the research was conducted in the absence of any commercial or financial relationships that could be construed as a potential conflict of interest.
